# Modified Huangqi Shengmai Yin enhances ruminal microbiome and metabolites activity in dairy cows with subclinical mastitis

**DOI:** 10.3389/fvets.2025.1631756

**Published:** 2025-08-08

**Authors:** Chenyibo Zhang, Baoling Zhang, Yuqiong Li, John P. Kastelic, Xiaoping Li, Xiaofang Tong, A. Yao, Chuang Xu, Bo Han, Jian Gao

**Affiliations:** ^1^Department of Clinical Veterinary Medicine, College of Veterinary Medicine, China Agricultural University, Beijing, China; ^2^Institute of Animal Science, Ningxia Academy of Agricultural and Forestry Sciences, Yinchuan, China; ^3^Faculty of Veterinary Medicine, University of Calgary, Calgary, AB, Canada; ^4^Animal Health Supervision Institute, Lincang, China

**Keywords:** subclinical bovine mastitis, Huangqi Shengmai Yin, microbiome structure, metabolites activity, dairy cows

## Abstract

**Introduction:**

Bovine mastitis, especially subclinical mastitis (SCM), with minimal clinical signs, is detrimental due to its resistance to treatment, recurrence, and substantial economic impact on global dairy industry. The modified form of Huangqi Shengmai Yin (HSY), classical traditional herbal medicine renowned for its effects in antimicrobial and circulatory-enhancing and thus beneficial for subclinical mastitis, has been developed for treatment attempt of SCM, yet its therapeutic effect and mechanism remains unclear. This study aims to investigate the therapeutic effects of mHSY on SCM in cows, and elucidate its potential therapeutic mechanism.

**Methods:**

In this study, mHSY was given orally to cows with SCM. After a 3-day treatment regimen, the therapeutic effects were evaluated. 16S diversity sequencing and metabolomics were used to elucidate the therapeutic mechanism of HSY.

**Results:**

The SCM was significantly alleviated after the 3-day treatment with HSY. In cows infected with SCM, there were significant alterations in rumen fluid microbiota, particularly proportions of *Enterobacter*, *Desulfovibrio*, and *Flavonifractor*, implying a pivotal role for these bacteria in SCM. Furthermore, the therapeutic potential of HSY is linked to improving the proportion of beneficial bacteria (e.g., *Succinivibrionaceae_UCG-001*) and re-establishing a balanced ruminal bacterial profile. Modulation of fatty acid and amino acid metabolism, as evidenced by changes in metabolite profiles, is a critical aspect of SCM and can be markedly ameliorated with mHSY administration.

**Conclusion:**

mHSY shows significant inhibitory effects on SCM, which may be attributed to regulating ruminal microbiota and metabolic pathways *in vivo*.

## 1 Introduction

Clinical mastitis, which has a high incidence and numerous causes, is one of the main diseases affecting the global dairy industry (1). Mastitis in dairy cows is attributable to various factors, including microbial infections (bacterial, fungal, mycoplasma, and viral), environmental factors (hygiene, feed, temperature, humidity, etc.), anthropogenic factors (mechanical injuries, milking stress, improper feeding management, etc.), and the cow’s own factors (e.g., age, lactation, feed, milk yield, and lactation period) (2). Subclinical mastitis, with minimal clinical signs and a high recurrence rate, also poses a serious threat and causes substantial economic losses for dairy farms (3). Traditional treatments rely on large amounts of antibiotics for lactating cows with clinical mastitis and at drying off. However, bacteria with increasing resistance to a wide spectrum of antibiotics are an impetus for effective and economical therapeutic regimes for subclinical mastitis.

Traditional Chinese Medicine, renowned for its distinct mechanisms and relatively low cost, is gaining momentum in modern pharmacy research. Numerous studies have substantiated the profound therapeutic efficacy TCM for treating diseases through regulation of immune and metabolic systems. Consequently, this approach is posited to mitigate overuse of antibiotics and reduce emergence of antimicrobial resistance (4–6).

Huangqi Shengmai Yin is a classical formula derived from Traditional Chinese Medicine, composed of *Astragalus*, pilose asiabell root, dwarf lilyturf root, magnolia vine fruit, and southern magnolia vine fruit. Officially documented in the Pharmacopeia of the People’s Republic of China, it is renowned for its effects in tonifying qi, generating body fluids, consolidating yin, and arresting sweating, traditionally used to treat conditions arising from dual deficiency of qi and yin, such as fatigue, palpitations, and shortness of breath (7). *Astragalus*, the principal herb in the formula, is sourced from the root of the leguminous plant *Astragalus membranaceus* (Fisch.) Bge.var.*mongholicus* (Bge.) *Hsiao*. It contains several bioactive compounds, including formononetin, astragalosides, and astragalus polysaccharides, which have been reported for their anti-inflammatory, antioxidant, and intestinal microbiota-modulating properties (8–10). Similarly, bioactive constituents such as polysaccharides, lignans, and triterpenoid saponins found in pilose asiabell root, dwarf lilyturf root, magnolia vine fruit, and southern magnolia vine fruit contribute to physiological homeostasis and cardioprotection by modulating immune responses and metabolic pathways (11–13). Previous studies confirmed heart-nourishing and immunological regulation functions of HSY, and it has been used to treat heart diseases like myocarditis, heart failure, and coronary heart disease in humans and pigs (14). Building upon its classical foundation, a modified preparation of HSY (commercially named as Ruyankang) was developed to address mammary gland disorders, with a modified formula comprising Chinese angelica root, *Astragalus* root, honeysuekle flower, forsythia fruit, Radix Trichosanthis, Mongolian Snakegourd Root, *Viola yedoensis* Makino, *Houttuynia cordata* Thunb., *Taraxacum mongolicum*, and *Glycyrrhiza uralensis* Fisch. From a traditional medical perspective, this reformulation shifts mHSY’s focus from qi and yin tonification to heat-clearing, detoxifying, blood activating, and stasis removing, namely antimicrobial and circulatory-enhancing (12, 15–18).

Arguably, subclinical mastitis could be related to immune system malfunction or perhaps affected by diet. Consequently, Chinese veterinary researchers are exploring the potential of HSY for treating bovine mastitis, especially subclinical mastitis. Previous research correlated the therapeutic function of HSY components with their effects on gut microbiome; the association between rumen microbiome and metabolome has been verified, but few studies have elucidated the relationship between HSY treatment and microbiome or metabolome (19, 20). In this study, mHSY was giving orally order to assess its therapeutic effects on SCM, with multi-omics used to elucidate underlying mechanisms.

## 2 Materials and methods

### 2.1 Experimental design and sample collection

The mHSY used in this study was jointly screened by research groups led by Li Yuqiong and Gao Jian. A suspension was prepared by mixing the powdered drug, at the specified dose (5 g/kg for each cow each day, from unpublished research of Li) with hot water, and then administered through a gastrostomy tube to subjects at 8 AM, daily for 3 days. This study was conducted on an intensively managed dairy farm in Ningxia Province, China with over 1,000 lactating cattle.

Based on milk somatic cell counts (SCC ≥ 2 × 10^5^ cells/mL), California mastitis test (CMT) results (weakly positive or positive), and udder clinical signs, 6 mid-lactation Holstein dairy cows with or without SCM were selected as the treatment group (T group) and control group (C group), respectively. Routine Dairy Herd Improvement (DHI) testing was performed by the local DHI testing center, and DHI samples were collected on Day 0 and Day 6. CMT scoring criteria followed the criterion of previous study (21), and SCC values from DHI report were converted to somatic cell scores (SCS) using the standard formula validated in dairy mastitis studies (22):


$$\mathrm{SCS}=\log_{2}\;(\mathrm{SCC}/100,000)+3$$

All cows received the same total mixed ration (TMR) and were housed in free-stall barns with *ad libitum* access to feed and water. The TMR was provided 3 times daily (07:30, 13:30, and 19:30). Samples of rumen fluid were collected on Day 0, followed by a 3-day mHSY administration. The cows were maintained on a standard diet throughout the experiment and had *ad libitum* access to water. Rumen fluid was sampled again on Day 6. Before sampling, cows were subjected to 12 h fasting to minimize dietary interference with metabolites. After local anesthesia was induced by subcutaneous injection of 2% procaine solution at a dosage of 0.1 mL/kg body weight around the rumen puncture site, rumen fluid samples (~ 10–20 mL) were collected through rumen puncture. In addition, ~ 20–30 mL of milk was collected from each cow (samples were a composite of milk from all 4 quarters) into sterile containers, and immediately cryopreserved at −80°C to maintain compositional stability. All samples were preserved in liquid nitrogen and subsequently analyzed via 16S rDNA sequencing and metabolomics.

### 2.2 DNA extraction, amplification and sequencing

Extraction of DNA from fecal and rumen fluid samples was performed using HiPure Stool DNA Kits (D3141, Guangzhou Meiji Biotechnology Co., Ltd., China) according to the kits’ instructions. Briefly, 150–200 mg of sample was transferred to a 2 mL tube and 1.2 mL of Buffer SSL was immediately added to the sample, followed by vortexing for 1 min at maximum intensity to break up the sample. After 10 min in a water bath at 70°C, the sample was vortexed for 15 s, then centrifuged at ≥14,000 g for 10 min at room temperature. Thereafter, 250 μL of supernatant was transferred to a new 1.5 mL tube.

To extract DNA, 20 μL of Proteinase K and 250 μL of Buffer AL supernatant were added to the sample, followed by gentle inversion mixing for 10 times. The mixture was then incubated at 70°C for 10 min to ensure complete protein digestion. Then, 250 μL of anhydrous ethanol was added, and the mixture was inverted 10 times. The HiPure DNA Mini Column I was loaded into a 2 mL collection tube, and the sample mixture was transferred onto the column. The column was centrifuged at 10,000 g for 30–60 s to bind the DNA to the silica membrane. The effluent was discarded, and the column was placed back into the collection tube. Next, 500 μL of Buffer GW1 was added to the column, followed by centrifugation at 10,000 g for 30–60 s to wash the membrane. The filtrate was discarded, and the column was returned to the collection tube. A second wash was performed by adding 650 μL of Buffer GW2 to the column and centrifuging at 10,000 g for 30–60 s. The column was then transferred to a 1.5 mL centrifuge tube, and 50–200 μL of Buffer AE, preheated to 70°C, was added to the center of the membrane. After allowing the column to stand at room temperature for 2 min, it was centrifuged at 13,000 g for 1 min to elute the purified DNA. The purified DNA was then stored at −20°C.

Sample quality was measured using a NanoDrop (NanoDrop 2000, Thermo Fisher Scientific, United States). Samples were amplified using a PCR instrument, using the following primers: CCTACGGGGNGGCWGCAG; 806R: GGACTACHVGGGTATCTAAT; 799F: AACMGGATTAGATACCCKG; 1193R: ACGTCATCCCCACCTTCC; Arch519F: CAGCMGCCGCGGGTAA; and Arch915R: GTGCTCCCCCGCCAATTCCT. Library quality testing was performed using the ABI StepOnePlus Real-Time PCR System (Life Technologies, United States), and up-sequencing was done using PE250 mode pooling with a Novaseq 6000 (NovaSeq6000 S2 Reagent Kit v1.5, Illumina, United States).

### 2.3 Data processing, annotation and statistical analysis

Amplicons were evaluated with 2% agarose gels and purified using AMPure XP Beads (Beckman, CA, United States) according to the manufacturer’s instructions. Sequencing libraries were generated using Illumina DNA Prep Kit (Illumina, San Diego, CA, United States) following manufacturer’s recommendation. Library quality was assessed with ABI StepOnePlus Real-Time PCR System (Life Technologies, Foster City, CA, United States). At the end, 2 × 250 bp paired-end reads were generated by sequencing on the Novaseq 6,000 platform. Raw reads were deposited into the NCBI Sequence Read Archive (SRA) database (Accession Number: SRR32089890 – SRR32089913). Raw reads were further filtered according to the following rules using FASTP (Version 0.18.0) to get high-quality clean reads (23). Paired reads were overlapped as raw tags using FLASH (version 1.2.11) with a minimum overlap of 10 bp and mismatch error rates of 2% (24). Noisy sequences of raw tags were filtered under specific filtering conditions to obtain high-quality clean tags (25) that were clustered into operational taxonomic units (OTUs) of ≥ 97% similarity using UPARSE (version 9.2.64) pipeline. All chimeric tags were removed using the UCHIME algorithm, and finally effective tags were obtained for further analysis.

Within each cluster, the tag sequence with the highest abundance was selected as the representative sequence (26, 27). Representative OTU sequences or ASV sequences were classified into organisms by a naive Bayesian model using RDP classifier (version 2.2) based on SILVA database (version 138.1) or UNITE database (version 8.3), with confidence threshold value of 0.8 (28, 29). Abundance statistics of each taxonomy were visualized using Krona (version 2.6) (30). The stacked bar plot of the community composition was visualized in the R project ggplot2 package (version 2.2.1). Pearson correlation analysis of species was calculated in the R project psych package (version 1.8.4). Species comparison between groups was calculated by Welch’s *t*-test and Wilcoxon rank test in the R project Vegan package (version 2.5.3). Biomarker features in each group were screened by LEfSe software (version 1.0), random forest package (version 4.6.12) in the R project, pROC package (version 1.10.0) in the R project, and lands package (version 2.0-1) in R project. Chao1, ACE, Shannon, Simpson, Good’s coverage, and Pielou’s evenness index were calculated in QIIME (version 1.9.1). Alpha index comparison among groups was computed by Tukey’s HSD test and Kruskal-Wallis H test in R project Vegan package (version 2.5.3). Jaccard and Bray-Curtis distance matrix calculated in R project Vegan package (version 2.5.3). FAPROTAX database (Functional Annotation of Prokaryotic Taxa) and associated software (version 1.0) were used for generating ecological functional profiles of bacteria (31).

### 2.4 Metabolite extraction and LC–MS/MS detection

For ruminal fluid and milk samples, ~ 100 mg of each sample was ground under liquid nitrogen and weighed. Subsequently, 1 mL of a pre-cooled methanol-acetonitrile-aqueous solution (v/v, 2:2:1) was added, and the mixture vortexed to ensure thorough mixing. Samples were then subjected to low-temperature ultrasound treatment for two 30 min intervals, followed by a 60-min static incubation at −20°C. After incubation, samples were centrifuged at 14,000 g for 20 min at 4°C, and the supernatant (equivalent to 50 mg of original sample) was collected. The supernatant was dried under vacuum, and mass spectrometry (MS) analysis was performed after reconstituting dried samples with 100 μL of an acetonitrile-water mixture (acetonitrile: water = 1:1, v/v) and vortexing. For further analysis, samples were re-dissolved with 100 μL of acetonitrile-water solution (acetonitrile: water = 1:1, v/v), vortexed, and centrifuged at 14,000 g for 15 min at 4°C, with supernatant used for subsequent analysis. Quality control (QC) samples were prepared by pooling equal volumes from samples to be tested. These QC samples were used to assess instrument status, to balance the chromatography-mass spectrometry system before injection, and to evaluate system performance throughout the experiment.

### 2.5 Metabolomics data preprocessing, annotation and analysis

Positive ion mode (POS) and negative ion mode (NEG) were both used to detect metabolites, improving metabolite coverage and the detection effect. In subsequent data analyzes, positive and negative ion models were analyzed separately. QC samples are usually used for quality control when the study of metabolomics is based on mass spectrometry. Theoretically, QC samples are the same. However, there could be systematic errors in sample extraction, detection or analysis, which would lead to differences among QC samples.

Principle component analysis (PCA) was performed by R language models (v2.18.1). Orthogonal partial least squares discriminant analysis (OPLS-DA) is a supervised dimensionality reduction method in which class memberships are coded in matrix form into Y to better distinguish the metabolomics profiles of two groups by screening variables correlated with class memberships. OPLS-DA was applied in comparison groups using R package ropls.^1^ A variable importance in projection (VIP) score of PLS model was applied to rank metabolites that were best distinguished between the 2 groups.

To understand regulation of differential metabolites, fold changes of abundance between 2 groups were further calculated to draw the volcano plot. Kyoto Encyclopedia of Genes and Genomes (KEGG) is the major public pathway-related database that includes genes and metabolites. Metabolites were mapped to KEGG metabolic pathways for annotation and enrichment analysis. Pathway enrichment analysis identified significantly enriched metabolic pathways or signal transduction pathways in differential metabolites compared to the whole background (32, 33).

### 2.6 Statistical analyzes

The *t*-test and VIP were used as a univariate analysis for screening differential metabolites. Metabolites with a *p*-value of *t*-test < 0.05 and VIP ≥ 1 were considered differential metabolites between the 2 groups. The SCS and SCC data are presented in the form of mean ± SD. Milk components and SCS data of the 2 groups of cows were analyzed using one-way ANOVA and Student’s *t*-test with SPSS Statistics 22 (IBM, Chicago, United States). Significance was declared at *p* < 0.05, and 0.05 < *p* < 0.10 represented a tendency.

## 3 Results

### 3.1 mHSY mitigated bovine subclinical mastitis and altered milk components

**Figure 1:**
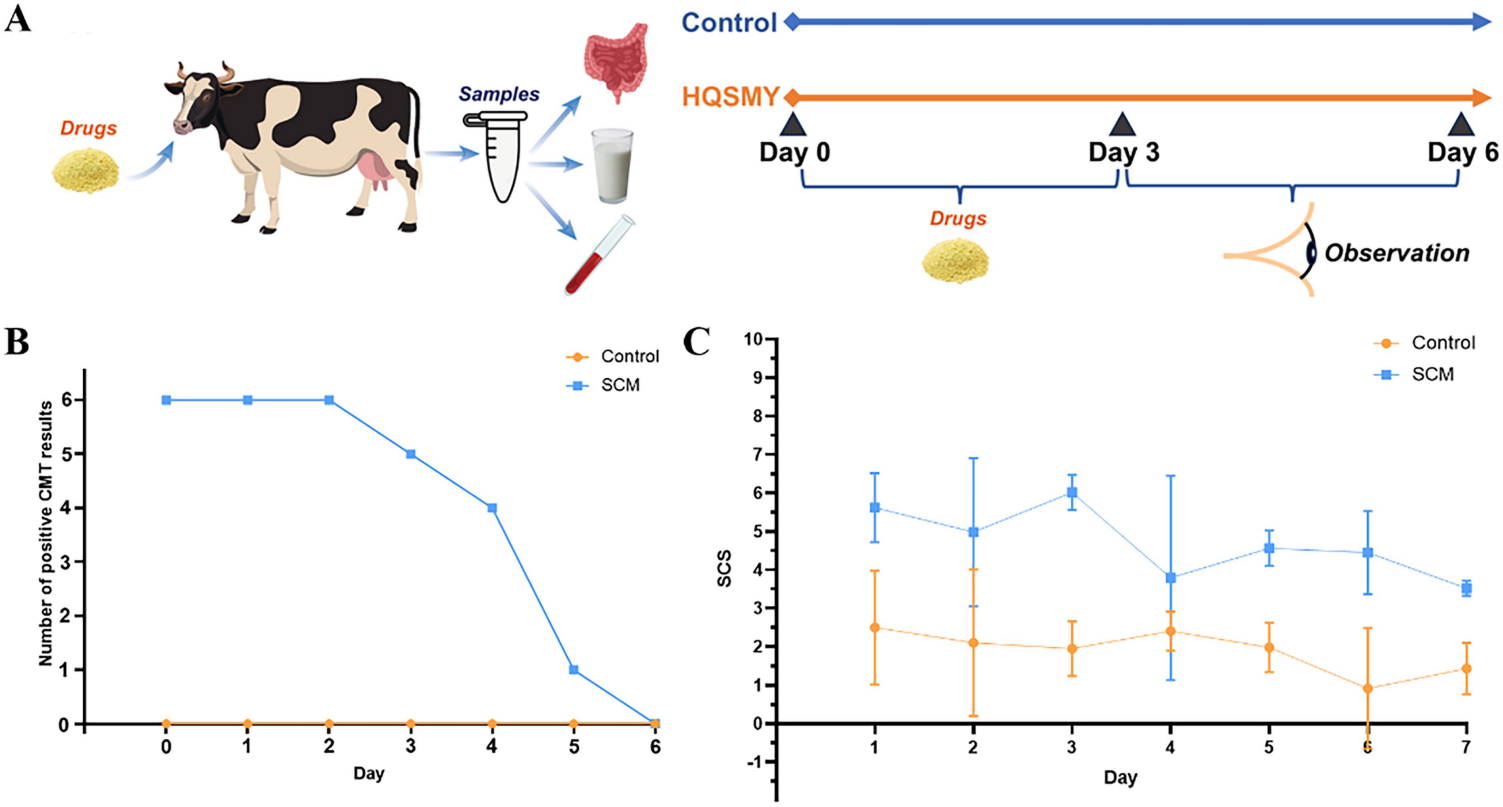
Cow with subclinical mastitis given a Traditional Chinese Medicine (mHSY). **(A)** Schematic diagram of the experimental procedure. **(B)** CMT test results. **(C)** Milk somatic cell count test results. All results are mean ± SD (*n* = 6 per group).

Six healthy cows and 6 SCM cows were selected for the experiment (Figure 1A). Both groups were given mHSY for 3 consecutive days, followed by an additional 3 days of observation. Samples of rumen fluid were collected on Day 0 and Day 6 for analysis of bacterial diversity and metabolomic profiling. Concurrently, the CMT test and milk somatic cell count were performed daily from Day 0 to Day 6.

The number of CMT-positive cows had minimal change during the initial 2 days and a slight decline from Day 2 to Day 4 (Figure 1B). However, a marked decrease was observed in cows with SCM on Day 5. In contrast, the number of presumably healthy cows remained consistent throughout the experiment. The curve of milk SCC had a similar pattern, decreasing from > 75 × 10^4^ cells/mL to ~ 25 × 10^4^ cells/mL, with a minor peak on Day 2. The SCC of healthy cows was consistently < 25 × 10^4^ cells/mL (Figure 1C).

Variations in milk composition were also analyzed through one-way ANOVA and Student’s *t*-test (Table 1). Milk fat and urea concentrations were not different (*p* > 0.05) between healthy and SCM cows, both prior to and after mHSY treatment. Conversely, there was a significant disparity between the 2 groups in milk protein percentage and SCC on Day 0; however, by Day 6, these parameters were not different between the 2 groups (*p* < 0.05). Interestingly, the percentage of lactose in milk exhibited an inverse trend, with no significant difference on Day 0, yet a divergence between the 2 groups was noted on Day 6 (p < 0.05).

**Table 1:** Milk composition in healthy and SCM cows before and after Traditional Chinese Medicine (mHSY) treatment.

Items	Experimental treatments				SEM	*P*-value
	C-D0^1^	C-D6	T-D0^2^	T-D6		
	(*n* = 6)	(*n* = 6)	(*n* = 6)	(*n* = 6)		
Milk fat (%)	2.01	1.19	1.75	1.52	0.17	0.637
Milk protein (%)	3.20^a^	3.24^a^	3.71^b^	3.23^ab^	0.12	0.049
Lactose (%)	4.13^ab^	4.21^a^	3.95^b^	3.90^b^	0.07	0.038
Milk SCC^3^ (×10^3^/mL)	99.17^b^	36.67^c^	717^a^	144.5^b^	157.45	<0.001
Urea (mg/dL)	6.15	8.41	8.36	5.84	0.69	0.088

1 C = healthy cattle.

2 T = cattle with subclinical mastitis.

3 SCC = somatic cell count.

^a,b,c^superscripts of the significant difference between different treatment groups in the same row (*p* < 0.05), different letters indicate significant difference, and the same letter indicates no significant difference.

### 3.2 mHSY reversed the SCM-induced negative impact on the richness, diversity, and composition of the rumen microbial community

**Figure 2:**
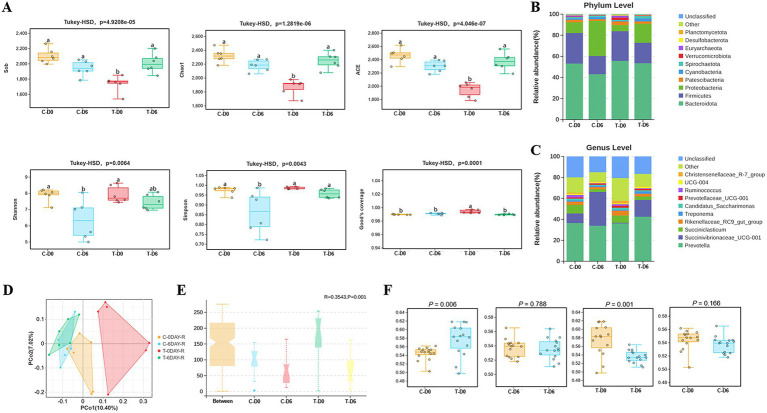
Alpha and beta diversity of the microbiota between healthy and SCM cows before and after Traditional Chinese Medicine (mHSY) treatment. **(A)** Alpha diversity indices and Good’s coverage of rumen fluid microbiomics in dairy cows, analyzed by Tukey-HSD analysis (Sob, Chao1, ACE, Simpson, Shannon, Good’s coverage). **(B,C)** Microbiota composition of rumen fluid at phylum level **(B)** and genus level **(C)**. **(D)** Principal co-ordinates analysis (PCoA) of ruminal microbiota based on an unweighted unifrac of OTUs. **(E)** Beta diversity results analyzed by Anosim based on an unweighted unifrac of OTUs. **(F)** Beta diversity results analyzed by Welch’s *t*-test. C, healthy cattle; T, cattle with subclinical mastitis.

A total of 6,193,093 high-quality 16S rRNA gene sequences were obtained from 24 rumen fluid samples, with Good’s coverage of 99% across all samples. Rarefaction curves indicated a gradual plateau in the increase of species numbers and diversity indices with the rise in the number of reads sampled, suggesting that sequencing data were adequate to capture the majority of microbial diversity. Significant differences were observed between healthy and SCM groups on Day 0 in the Sobs, Chao1, and ACE indices (Figure 2A), with no significant differences between the 2 groups in Shannon or Simpson indices. After mHSY treatment, Sobs, Chao1, and ACE indices of SCM cows had significant increases, whereas those of the control group remained stable. In contrast, Shannon and Simpson indices for healthy cows were significantly decreased. On Day 6, the Simpson index for SCM cows was significantly higher compared to that of healthy cows. With the exception of the SCM group on Day 0, there were no significant differences among groups in Good’s coverage.

After sequence clustering and quality filtering, a total of 11,005 OTUs with > 97% similarity were identified. Using the Unweighted Pair Group Method with Arithmetic Mean (UPGMA) for taxonomic analysis of similar OTU representative sequences, 28 phyla and 338 genera of bacteria were identified. At the phylum level, predominant groups before and 3 days post-administration were Bacteroidota (51.29% ± 5.62%), Firmicutes (23.45% ± 5.99%), Proteobacteria (16.84% ± 12.08%), Patescibacteria (2.39% ± 0.76%), and Cyanobacteria (1.71% ± 0.97%) (Figure 2B). At the genus level, the most abundant microorganisms included *Prevotella* (37.20% ± 3.71%), *Succinivibrionaceae_UCG-001* (14.53% ± 13.43%), *Succiniclasticum* (5.24% ± 2.74%), *Rikenellaceae_RC9_gut_group* (3.06% ± 1.12%), and *Treponema* (1.44% ± 0.61%) (Figure 2C).

Principal coordinates analysis (PCoA) of rumen microbial communities was conducted based on the Unweighted UniFrac distance algorithms (Figure 2D); differences were assessed using the Anosim test, with R acting as a statistic. In Figure 2E, an R > 0 indicated that the intra-group distances were smaller than inter-group distances, which validated groupings. Furthermore, microbial composition differed (*p* = 0.001) between control and mHSY treatment groups, with Welch’s *t*-test used to provide more detailed comparisons. A difference in beta diversity between the control group and the SCM group (*p* < 0.05) implied that the etiology of SCM may be rooted in alterations within the rumen microbiome. After mHSY treatment, the lack of a difference (*p* > 0.05) between the control group before and after mHSY administration implied that mHSY was safe, as it did not appear to disrupt the original rumen microbiome. However, after mHSY administration, there was a difference (*p* < 0.05) in microorganism diversity within the SCM group. Moreover, the lack of a difference between the 2 groups on Day 6 (*p* > 0.05) implied that mHSY significantly restored the impaired rumen microbiome induced by SCM toward a healthier state (Figure 2F).

### 3.3 mHSY altered ruminal bacteria composition at phylum and genus levels

**Figure 3:**
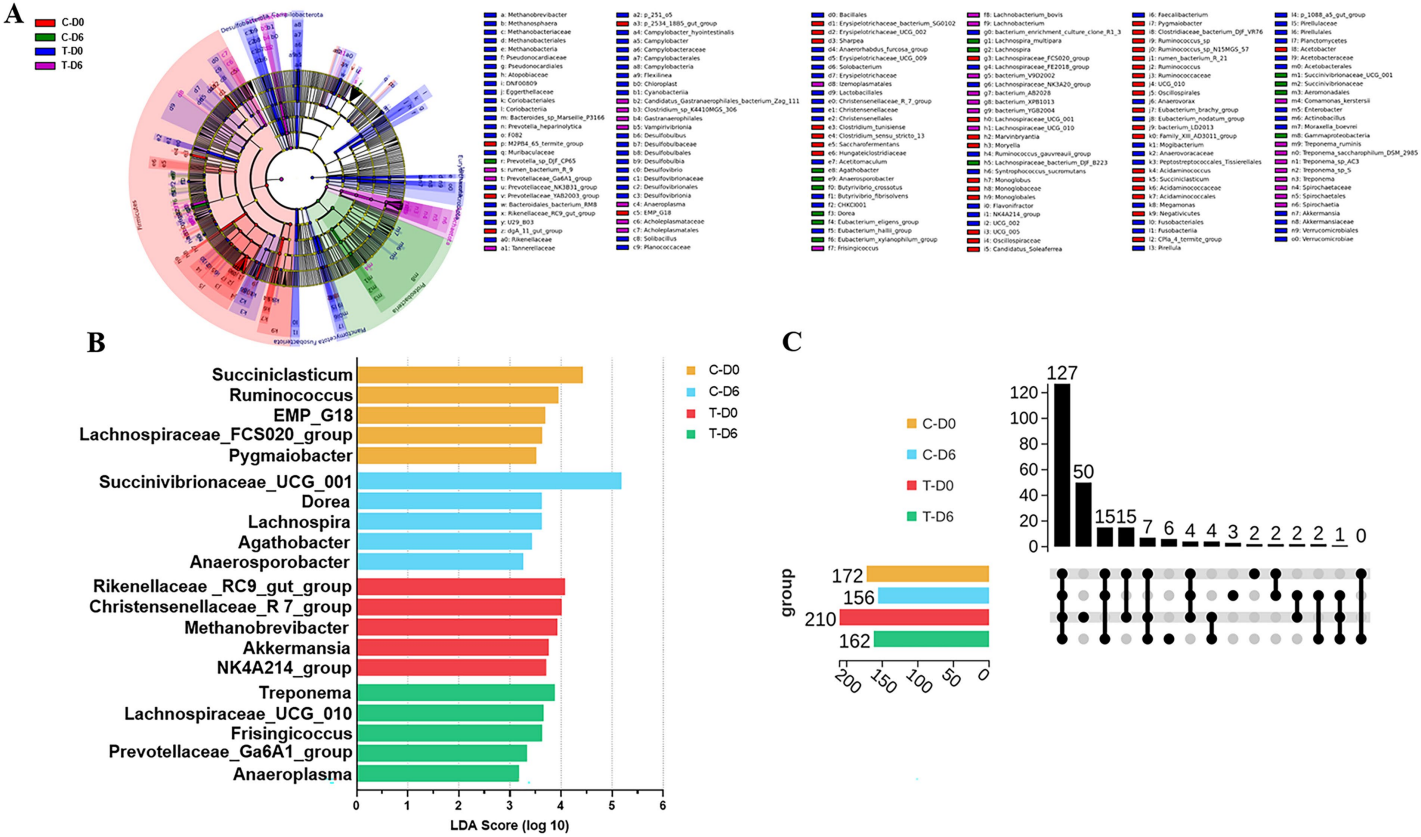
**(A)** Cladogram indicating linear discriminant analysis effect size (LEfSe) analysis of different ruminal microbiota between healthy and subclinical mastitis (SCM) cows before Traditional Chinese Medicine (mHSY) treatment. **(B)** Linear discriminant analysis (LDA) score plot indicating the effects of top 5 microbiota on difference on genus level between healthy and SCM cows before mHSY treatment. **(C)** Upset diagram of shared ruminal bacteria taxa between healthy and SCM cows before and after mHSY administration.

A non-parametric Kruskal-Wallis sum-rank test was used to detect differences in ruminal bacteria between the 2 groups. Significantly diverse microbes (genus level) were assembled in 14 phyla, including Firmicutes, Bacteroidota, Proteobacteria, Planctomycetota, Euryarchaeota, Actinobacteriota, Desulfobacterota, etc. Significantly different bacteria at the genus level were filtered with linear discriminant analysis effect size (LEfSe), as shown in Figure 3A, with the top 5 on the Linear discriminant analysis (LDA) score listed in Figure 3B.

The upset diagram displayed the number of unique microorganisms on the genus level (Figure 3C). A total of 127 genera were shared by the control and SCM groups before and after mHSY administration, which was subjected to correlation analysis with ruminal metabolites. SCM cows harbored 50 unique genera, whereas healthy cows had only 2 distinctive genera, with no genus shared by the 2 groups. Therefore, there was a dramatic difference between SCM cows and healthy individuals. However, after mHSY treatment, the healthy group had 3 unique genera and shared 2 unique genera with the SCM group, whereas 6 unique genera were exclusive to the SCM group. The rumen microbiome of the SCM group at Day 0 was obviously distinct from that of the other groups; however, mHSY treatment appeared to bridge this gap.

### 3.4 Metabolic profiling of ruminal fluid samples

**Figure 4:**
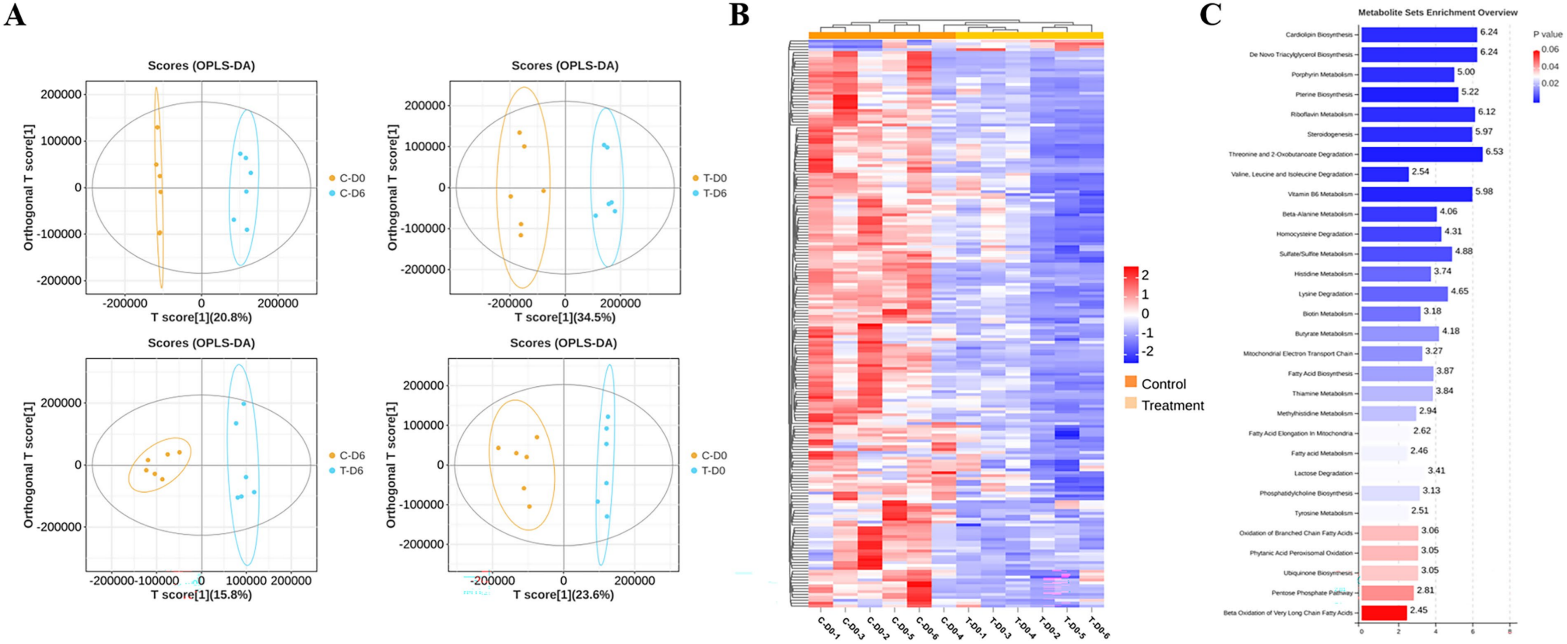
Ruminal metabolites were analyzed with OPLS-DA, heatmap, and MSEA pathway enrichment. **(A)** OPLS-DA of metabolic sets between healthy and subclinical mastitis (SCM) cows before and after Traditional Chinese Medicine (mHSY) administration. **(B)** Heatmap of significantly differential metabolites between healthy and SCM cows before mHSY administration. **(C)** MSEA pathway enrichment of significantly differential metabolites between healthy and SCM cows before mHSY administration.

An untargeted metabolomics analysis based on liquid chromatography-mass spectrometry (LC–MS) technology was used to examine metabolite profiles in rumen fluid. Through overlapping comparison, the response intensity and retention time of each chromatographic peak were essentially overlapped, which reflected reliability of data quality. After filtering and optimizing, a total of 23,528 ruminal metabolites (13,220 and 10,308 in positive and negative ion modes, respectively) were identified from the 24 samples. An orthogonal partial least squares discriminant analysis (OPLS-DA) was conducted based on a supervised multivariate statistical analysis method to reflect overall differences among various groups and variability within sample groups. Rumen fluid samples were clearly separated according to their metabolic profiles across groups, as evidenced by the OPLS-DA score plot in the merging ion mode (Figure 4A). The heat map, which displays relative abundance of various metabolites and their top 30 enriched pathways based on the adjusted *p*-value, for samples from healthy and SCM cows in merging ion mode, is presented in Figures 4B,C.

### 3.5 Significantly different metabolites in ruminal fluid of cows given mHSY

**Figure 5:**
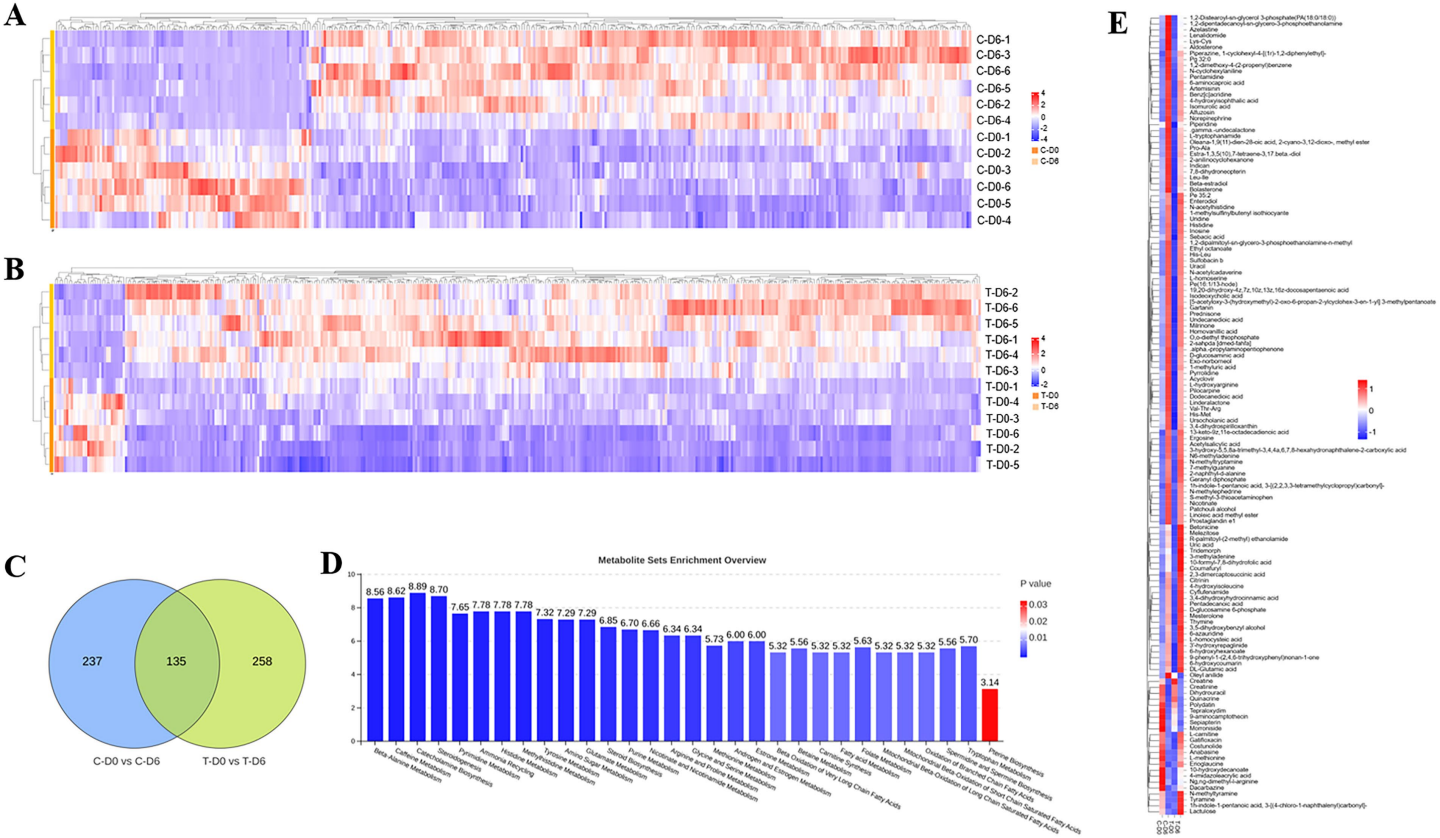
Significantly differential metabolites between healthy and subclinical mastitis (SCM) cows before and after Traditional Chinese Medicine (mHSY) administration. **(A,B)** Significantly differential metabolites of healthy cows **(A)** and SCM cows **(B)** before and after mHSY administration (VIP > 1, *p* value <0.05, 0.67 < FDR < 1.5). **(C)** Venn diagram of significantly differential metabolites before and after mHSY treatment. **(D)** MSEA pathway enrichment of significantly differential metabolites before and after mHSY treatment. **(E)** Heatmap of significantly differential metabolites among groups.

Differential ruminal and mammary metabolites with a VIP value of >1, a fold change of >1.5 or <0.67, and *p* < 0.05 were identified among the control group and SCM group before and after mHSY administration. Ionic strengths from merging mode of metabolites between groups are shown in Figures 5A,B. A total of 372 and 393 metabolites in the rumen fluid of healthy and SCM cows, respectively, underwent significant changes during mHSY treatment, with 135 shared metabolites used for further analysis (Figure 5C). After mHSY treatment, expression of most ruminal metabolites was upregulated in both groups; however, expression of Creatine, Creatinine, Dihydrouracil, Quinacrine, Polydatin, Tepraloxydim, 9-aminocamptothecin, Sepiapterin, and Morroniside was downregulated in both groups. Expression patterns of Oleyl anilide, L-carnitine, Gatifloxacin, Costunolide, Anabasine, L-methionine, Erioglaucine, 10-hydroxydecanoate, 4-imidazoleacrylicacid, Ng,ng-dimethyl-l-arginine, Dacarbazine, N-methyltyramine, Tyramine, 1 h-indole-1-pentanoic acid, 3-[(4-chloro-1-naphthalenyl)carbonyl]-, and Lactulose were divergent between healthy and SCM cows, with the majority except for Oleyl anilide decreasing in healthy cows but increasing in cows with SCM (Figure 5E). Metabolite Set Enrichment Analysis (MSEA) was conducted on rumen fluid metabolites from the Control group before and after mHSY treatment, as well as that from the SCM group, with the top 30 metabolites pathways sorted from highest to lowest according to FDR-adjusted *p*-value. The most significantly enriched pathways included amino acid metabolism, nucleotide metabolism, metabolism of cofactors and vitamins, and lipid metabolism are shown in Figure 5D.

### 3.6 Correlations between differential rumen microbiota, ruminal and mammary metabolites, and DHI

**Figure 6:**
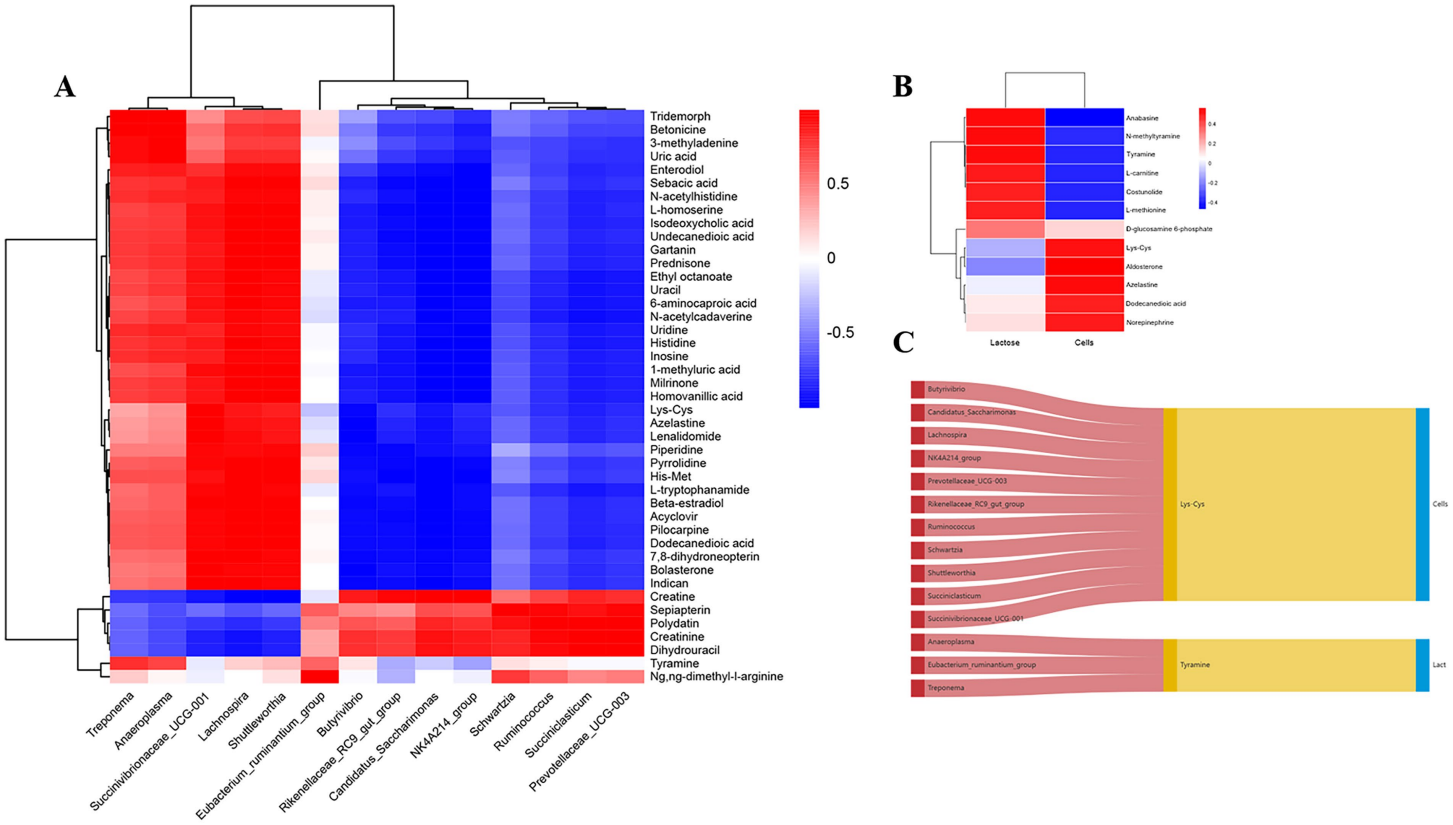
Correlation analysis between ruminal microbiome, metabolome and milk components before and after Traditional Chinese Medicine (mHSY) treatment. **(A)** Correlation heatmap of significantly differential ruminal bacteria and metabolites before and after mHSY treatment. **(B)** Correlation heatmap of significantly differential ruminal metabolites and milk components. **(C)** Sankey diagram of correlations among ruminal microbiome, metabolome and milk components.

Before analyzing the correlation between differential microbiota and ruminal and mammary metabolites, unknown differential metabolites lacking CAS codes, unclassified microbiota and those with a relative abundance < 0.1% were excluded, whereas only DHI indices that demonstrated significant differences were included in the analysis. The Pearson correlation coefficient model and genus-level heat maps were mapped (Figure 6A), including only metabolites and bacteria with an absolute Pearson correlation coefficient value >0.5 and ranking in the top100 by absolute value. The Pearson correlation coefficient model and corresponding correlation heat maps among ruminal and mammary metabolites are in Figure 6B. The Sankey diagram, depicting interrelationships among ruminal microbiota, ruminal and mammary metabolites, and DHI, was mapped (Figure 6C).

## 4 Discussion

Subclinical mastitis causes large milk production losses in the dairy sector worldwide. Unlike clinical mastitis, it presents a diagnostic challenge due to the lack of overt clinical signs (e.g., udder swelling and redness), and has a much higher prevalence than clinical mastitis. Furthermore, SCM can progress to clinical mastitis (34). Traditionally, antibiotic therapy has been the primary method for managing SCM; however, antibiotics may disrupt the rumen microbiome and can lead to other health disorders (35). Conversely, TCM, mainly composed of medical plants and minerals, usually have less side effects. Their beneficial effects on managing various diseases are achieved by modulating the animal’s physiological state and intestinal microbiota, thereby providing a holistic therapeutic approach. This character makes for their widespread application as dietary additives to enhance immunoregulation, anti-inflammation, anti-stress, and prevent underlying diseases in many species (8, 36, 37). Moreover, TCM promises to decrease antibiotic use, which is particularly vital if there is increasing antibiotic resistance (38–40). Therefore, TCM should improve animal welfare and address some environmental issues.

In recent years, the entero-mammary pathway theory has gained traction, with research increasingly focused on uncovering links between microbiomes and metabolomes in the mammary gland and gastrointestinal tract (41–43). Studies of SCM often examine shifts in the metabolome and microbiota of either the udder or intestine (44–46). Admittedly, SCM tends to lead to a divergent microbial landscape within the intestines, resembling the dysbiosis present in inflammatory bowel disease, which disrupts the intestinal microbiome (21, 47). Unlike monogastric animals, ruminants have a rumen—a fermentation chamber housing diverse microorganisms crucial for digestion. Within these populations, *Succiniclasticum*, *Prevotella*, *Treponema, Dorea, Lachnospiraceae* and *Ruminococcus* are regarded as beneficial due to their roles in breaking down cellulose, hemicellulose, starch, and proteins into short-chain fatty acids (SCFAs) and amino acids, which are energy sources for the host and milk precursors (48–50). The gastrointestinal barrier, vital to preventing endogenous pathogen and virulence factors from entering mammary gland through blood and thus avoid inflammation, also relies on sufficient SCFAs (21). In this study, mHSY promoted a shift toward commensal microbiota dominated by SCFA-producing bacteria in both groups. Critically, in cows with SCM, this microbial restructuring was accompanied by significantly reduced SCC. This improvement in udder health is proposed to mechanistically correlate with enhanced production of ruminal SCFAs through the mechanism mentioned above. Competitive exclusion of pro-inflammatory bacteria in SCM pathogenesis may also happened during this microbial alteration.

In association with alternations in ruminal microbial composition, levels of differential metabolites associated with energy and vitamin metabolism in both healthy and SCM cows were also observed after mHSY treatment, indicating changed energy metabolism and biosynthesis pathways in the rumen. Notably, mHSY appeared to reverse the downregulated amino acid metabolism observed in cows with SCM, such as methionine, histidine, tryptophan and arginine, essential for milk protein production (51). Besides, altered steroidogenesis, fatty acid metabolism, oxidation of branched-chain fatty acids and very long chain fatty acids revealed that mHSY may modulate energy supply and reproductive function in dairy cows (52). Androgen and estrogen metabolism was also significantly affected by mHSY, which may in turn regulate proliferation rates of mammary epithelial cells (53). Correlation analysis revealed that specific rumen bacteria, including *Treponem*a, *Succinivibrionaceae_UCG-001*, *Lachnospira*, and *Shuttleworthia*, were positively associated with metabolites such as uric acid, sebacic acid, and N-acetylhistidine, which are integral to amino acid, lipid, and nucleic acid metabolism (54, 55). Conversely, other bacteria like *Butyrivibrio*, *Ruminococcus*, and *Succiniclasticum* had negative correlations with energy metabolism, possibly due to niche competition among microbial populations (56). Furthermore, different microorganisms may get involved in diverse parts of the same pathway, thus leading to seemingly opposite functions in metabolism correlation (57). This suggests that different microorganisms may exert diverse and sometimes opposing effects on metabolic pathways, highlighting the complexity of rumen microbiota interactions. SCC in milk tends to correlate negatively with lactose concentration, contributing to the contradicted correlation in some ruminal metabolites (58). However, few studies have elucidated the mechanism underlying the correlation between the ruminal organisms and metabolites and milk components shown in our study. In addition, we inferred that the rumen metabolic profile and microbial structure in cows with SCM are closely linked to milk quality. mHSY had substantial potential in alleviating SCM and enhancing milk quality, likely through its role in modulating the ruminal microbiome and subsequently regulating metabolism. Further studies are needed to investigate effects of mHSY’s individual components on the ruminal microbiome, especially *Succiniclasticum*, to elucidate its pharmacological mechanisms and specific therapeutic target.

This study has several limitations that warrant consideration. Given the association between the rumen microbiome and mastitis, this study emphasized the rumen microbiome and metabolome when investigating SCM, aligning with findings from other bovine metabolic diseases (42). Community richness was significantly lower in rumen fluid of cows with SCM compared to healthy individuals, consistent with that of Zhu et al. (59). However, diversity levels between groups were similar, in contrast to Zhong et al. (60). Though overt clinical issues were not observed in this study, the observed ruminal alterations in healthy cows necessitate more comprehensive evaluation including longitudinal clinical monitoring of rumen fermentation function, metabolic profiles, and general health indicators to fully establish safety. The relatively small sample size may have reduced statistical power and introduced bias toward individual variability. Additionally, the absence of an untreated SCM control group limits our ability to definitively attribute the observed reductions in SCC and changes in ruminal parameters solely to the administration of mHSY. Future research should address these limitations through randomized controlled trials incorporating untreated and placebo control groups, systematic collection of comprehensive production data, and expanded sample sizes to validate these preliminary findings. It is important to note that this study primarily focused on SCC as the key indicator of SCM. This study was primarily designed to investigate the effects of mHSY on SCC and the ruminal microbiome and metabolome in SCM cows. As a result, production parameters such as individual milk yield before and after treatment were not systematically collected in this study. While the randomization process aimed to minimize bias, the absence of these potential confounding factors limits our ability to fully account for their influence on treatment response. Future studies incorporating comprehensive production records and detailed individual histories would be valuable to confirm and extend these findings. Moreover, long-term studies should be conducted to determine impacts of mHSY treatment on the recurrence rate and chronicity of subclinical mastitis, which would provide valuable insights into long-term efficacy and potential side effects of mHSY.

In summary, this study demonstrated the potential of mHSY, a TCM with efficacy in treating myocarditis, heart disease, and allergic shock, as a novel therapeutic agent for SCM in dairy cows (14). In this study, mHSY significantly alleviated SCM parameters in dairy cows, with no detectable adverse effects on SCC observed in healthy cows, confirming its favorable safety profile. We inferred that mHSY may have anti-inflammatory properties and protect the blood-milk barrier (9, 11, 61). mHSY has promising effects in modulating the ruminal microbial composition and metabolome, critical factors in the pathogenesis of SCM. In practical veterinary applications, mHSY has potential as an alternative or complementary approach to conventional antibiotic therapies by improving the overall health and productivity of dairy herds. The optimal dosing regimen, which remains to be determined through further studies, will be crucial in maximizing the efficacy and minimizing potential adverse effects of mHSY. Furthermore, validation of mHSY’s efficacy would be a valuable advance in management of SCM, contributing to improved animal welfare and enhanced productivity in the dairy industry.

## 5 Conclusion

This study was a comprehensive analysis of the gastrointestinal microbiome and associated metabolite alterations in dairy cows with SCM and their response to mHSY treatment. There were significant alterations in *Enterobacter*, *Desulfovibrio* and *Flavonifractor* populations in rumens of affected cows, implying a crucial role in disease pathogenesis. Furthermore, mHSY demonstrated therapeutic potential by normalizing the gastrointestinal microbiota. Additionally, there were significant shifts in lipid and amino acids metabolism, which are pivotal for the pathophysiology of subclinical mastitis and are substantially ameliorated with mHSY intervention. These findings underscored the remarkable efficacy of mHSY in managing SCM.

## Data Availability

The datasets presented in this study can be found in online repositories. The names of the repository/repositories and accession number(s) can be found at: https://www.ncbi.nlm.nih.gov/, SRR32089890 – SRR32089913.

## Footnotes

^1^ http://www.r-project.org/
